# Molecular and Genetic Characterization of Newly Released CIMMYT Inbred Maize Lines

**DOI:** 10.3390/plants14243866

**Published:** 2025-12-18

**Authors:** Haihong Fan, Jianghao Wang, Yuanyuan Yan, Quanguo Zhang, Liwei Wang, Liang Song, Jianfeng Wei, Xinhua Li, Dongmin Zhang, Jinjie Guo, Rui Guo, Wei Song

**Affiliations:** 1 Key Laboratory of Crop Genetics and Breeding of Hebei Province, Institute of Cereal and Oil Crops, Hebei Academy of Agriculture and Forestry Sciences, Shijiazhuang 050035, China; 2 State Key Laboratory of North China Crop Improvement and Regulation, Hebei Sub-Center for National Maize Improvement Center, College of Agronomy, Hebei Agricultural University, Baoding 071001, China; 3 Hebei Key Laboratory of Crop Cultivation Physiology and Green Production, Institute of Cereal and Oil Crops, Hebei Academy of Agriculture and Forestry Sciences, Shijiazhuang 050035, China

**Keywords:** CIMMYT maize lines, genetic diversity, population structure, heterotic groups

## Abstract

Tropical germplasm has accumulated a large number of genes adapted to a variety of adversities. In this study, we assessed the genetic diversity and population structure of 109 inbred maize lines newly released from the International Maize and Wheat Improvement Center (CIMMYT) in the last few years. The results indicated the following: (1) linkage disequilibrium (LD) analysis showed that tropical maize germplasms had a faster rate of LD decay, suggesting higher recombination rates and genetic diversity; (2) both the phylogenetic tree and structure analysis supported the classification of the material into three subgroups; (3) the results of the principal component analysis were consistent with the population structure analysis, further verifying the reliability of subgroup delineation; (4) the genetic distances between the tropical germplasms from groups 2 and 3 and the elite temperate inbred lines were relatively close, which is suitable for temperate germplasms improvement. The results can help us select suitable tropical germplasms and speed up the process of inbred line development and maize improvement.

## 1. Introduction

Maize (*Zea mays* L.) originated in Mexico, and it has been cultivated for many centuries [1]. About 9000years ago, maize originated independently from the Teosinte subspecies *parviglumis* [2], and the “secondary origin hypothesis” suggests that the old maize and the Teosinte subspecies *mexicana* had a hybridization around 6000 years ago [3]. As one of the world’s most important food crops, it plays a vital role in both the national economy and the whole society [1]. Tropical maize, in particular, is a globally significant crop, and its genetic diversity is crucial for addressing challenges related to climate change [4], pest and disease pressures, and food security [5]. Since its establishment in 1966, the International Maize and Wheat Improvement Center (CIMMYT) has dedicated efforts to optimizing maize production systems, with a strong emphasis on promoting the sustainable development of smallholder farmers in tropical and subtropical regions [5]. Germplasms from CIMMYT collections include over 32,000 maize accessions and a diverse array of wheat relatives, forming a critical reservoir of genetic diversity to cope with emerging threats in agriculture. Tropical maize germplasms, including CMLs, exhibit unique advantages in stress tolerance and adaptability [5,6]. A total of 65 favorable alleles of 108 previously identified drought-resistant candidate genes were found in CIMBL55, which may constitute the genetic basis for its excellent drought resistance [6]. The utilization of these germplasm resources has not only boosted crop yields across different ecological zones but also enhanced their resilience to abiotic stresses [4].

China is one of the major maize-producing regions in the world, ranking second in maize production, after the United States. With a history of maize cultivation spanning nearly 500 years [7], China has developed its unique resources through several rounds of resource introduction and improvement, strongly supporting the nation’s food security [8]. The Huanghuaihai region, also called China Summer Maize (CSM) ecological region, is one of China’s six major corn-producing areas, characterized by concurrent rainfall and heat, as well as frequent droughts and floods [9]. With climate change, both biotic and abiotic stresses are becoming increasingly prevalent in this region, necessitating the introduction of new germplasms to improve existing varieties. The utilization of tropical germplasm for maize material improvement is receiving increasing attention from breeders in this region [9,10].

Maize is a pioneer crop for heterosis utilization [11]. In heterosis breeding, genetically diverse inbred lines with superior combining ability are used to develop F1 hybrids that exhibit heterosis, specifically, greater stress tolerance, adaptability, and yield than their parents [12]. Heterotic groups are primarily classified through the integration of pedigree records, combining ability tests, and phenotypic evaluation [13]. Within the CMLs germplasm, lines have been categorized into two main groups—Group A and Group B, or Tuxpeño and non-Tuxpeño—based on kernel characteristics and combining ability [8,14]. However, these groups often exhibit overlapping genetic backgrounds and remain challenging to reliably distinguish [15]. Several studies have examined both Chinese germplasm broadly and germplasm from specific ecological regions [7,8,9]. Shu et al. [9] used 490 inbred lines collected from researchers in the Huanghuaihai region and indicated that the two predominant types of heterotic pattern in this region are M-Reid group × TSPT group, and X subgroup × Local subgroups.

With the development of molecular marker technology, genetic diversity analysis based on SNP markers provides new technical means for germplasm resources evaluation and utilization [16]. Wu et al. [5] identified clear population structure and divergence between temperate and tropical subgroups, with three adaptive clusters emerging: Lowland Tropical, Subtropical/Mid-altitude, and Highland Tropical. In a comparative study using DNA markers, Guo et al. [10] found that CIMMYT lines were mainly clustered by pedigree and ecological adaptation, rather than heterotic affinity. In contrast, U.S. Plant Variety Protection (ex-PVP) is clearly separated into the classical Stiff Stalk Synthetic (BSSS) and non-Stiff Stalk Synthetic (NSSS) heterotic groups. In China, heterotic group classification typically involves four to six major clusters, with specific heterotic patterns tailored to different agro-ecological zones. Using 269 widely deployed temperate inbred lines, Zhang et al. [7] identified seven heterotic groups through cluster and principal coordinate analyses, tracing shifts in breeding emphasis over decades. Similarly, Wu et al. [17] used 1015 SNPs, and 367 inbred lines from Chinese maize breeding programs were divided into five groups.

Tropical maize is a genetic treasure trove of resistant genes and favorable traits [6,16,18], for example, drought resistance and provitamin A content in the maize kernel. In this study, 109 tropical maize germplasms and 6 temperate elite inbred lines were collected, and their genetic variation structures were systematically resolved. In this study, we aim to (1) investigate the population structure and heterotic patterns of these newly released CML lines, (2) estimate the genetic relationships among the CMLs and elite temperate inbred lines, and (3) assess how CMLs could be utilized to improve temperate maize.

## 2. Results

### 2.1. Distribution and Statistical Analysis of SNP Markers on Chromosomes

**Figure 1:**
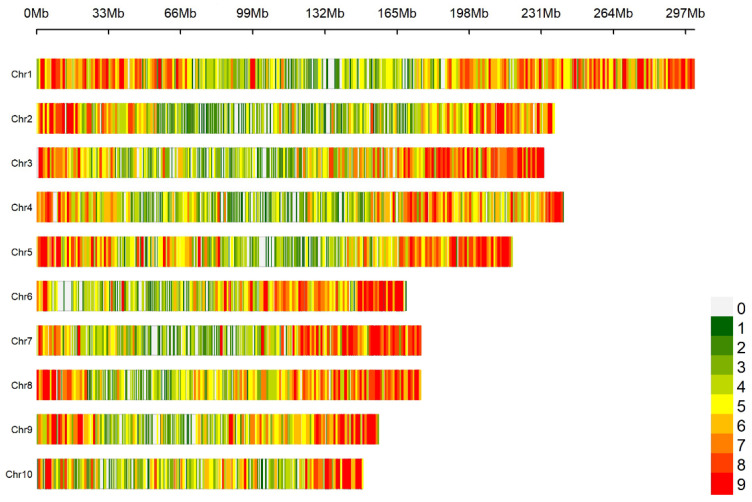
Density distribution of SNP markers on chromosomes.

A genome-wide scan based on 10 K SNP microarrays with a 1 Mb window sliding window analysis revealed a non-uniform distribution pattern of SNPs in the maize genome (Figure 1). A total of 10,005 SNPs were obtained. The distribution of SNP markers on all 10 maize chromosomes and the heatmap of marker density are shown in Figure 1. The SNP markers covered the whole genome and were evenly distributed on each chromosome. SNP markers covered the whole genome and were evenly distributed on each chromosome. Statistical analysis of the minor allele frequency, missing rate, heterozygosity rate, and Shannon index of 10,005 SNP marker sites showed that the mean of minor allele frequency (MAF), missing rate, heterozygosity rate, and Shannon index of the 10 chromosomes were 0.257, 0.027, 0.025, and 0.521, respectively, indicating that the genetic diversity of maize CML germplasms was rich. The missing rate and heterozygosity rate are both relatively low, indicating that the quality of the genotype data is quite high. The mean value of each index of SNPs in each chromosome had little difference (Table 1).

### 2.2. Heterozygosity and Missing Rates in 109 CML Germplasms

**Figure 2:**
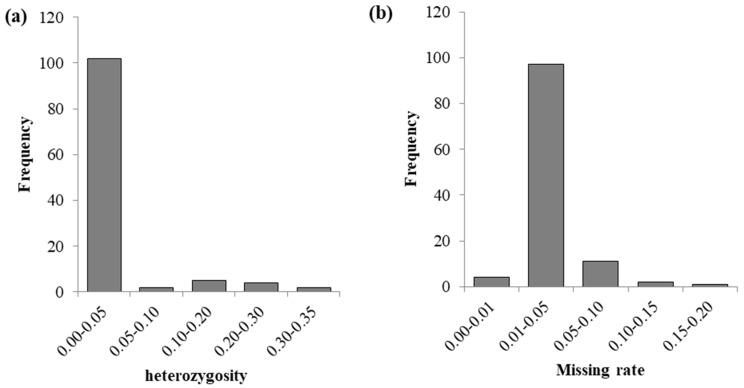
Distribution of heterozygosity and missing rates in 109 CML germplasms. (**a**) is the distribution of heterozygosity, and (**b**) is the distribution of the missing rate.

From the histogram (Figure 2), it can be seen that the heterozygosity is mainly concentrated between 0.01 and 0.05, and the missing rates are mainly concentrated between 0.01 and 0.05. Among the 109 CML accessions, the missing rates of 3 accessions (CML534, CML545, and CML541) are greater than 0.1, and the heterozygosity rates of 6 accessions (CML612B, CML538, CML523, CML540, CML603, and CML518) are greater than 0.2. Overall, the deletion rates and heterozygosity rates of these 109 CML accessions are relatively low.

### 2.3. Linkage Disequilibrium Analysis

**Figure 3:**
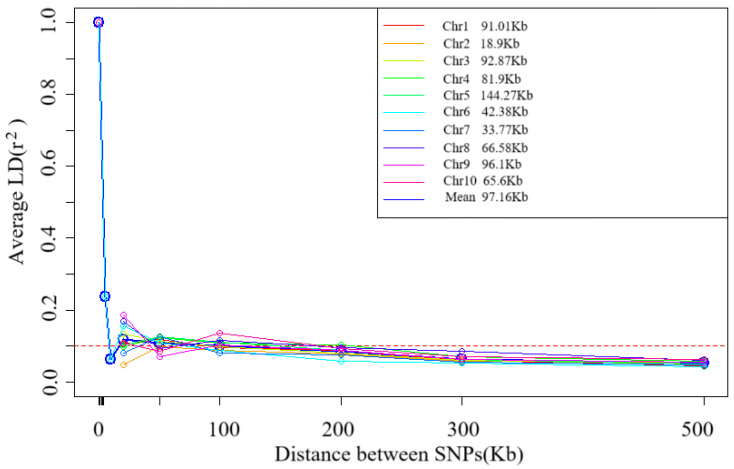
Trends of mean r^2^ over different intervals of LD decay distance of 10 chromosomes and total chromosomes.

Decay pattern of Linkage Disequilibrium (LD) with physical distance. The horizontal axis is the physical distance (in Kb), and the vertical axis is r-squared, a measure of LD strength. The data in the figure show that when r-squared decays to a threshold value of 0.1, the corresponding average physical distance is 97.16. The decay pattern of LD with the physical distance on 10 chromosomes (Chr1–Chr10) of 109 tropical maize germplasms was evaluated using Linkage Disequilibrium Decay (LD) analysis. The results showed significant heterogeneity in the decay distances across chromosomes (range: 18.90 Kb–144.27 Kb) with an average decay distance of 97.16 Kb when the LD intensity (r^2^) decayed to 0.1 (Figure 3). This variation reflects the inter-chromosomal recombination rate differences.

### 2.4. Structure Analysis

**Figure 4:**
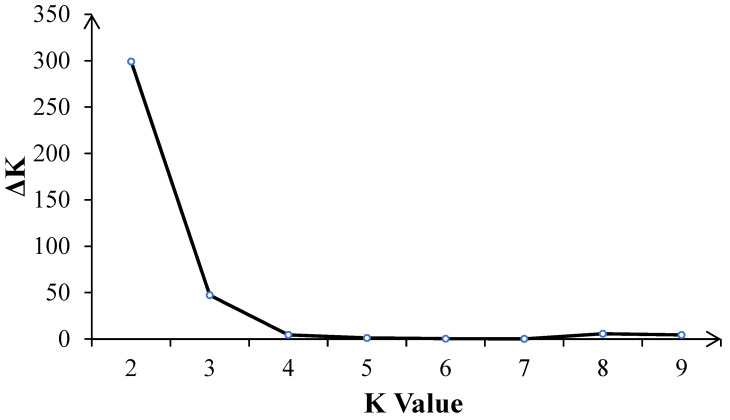
The number of inferred optimal populations (K) in the subset of 109 CMLs and 6 elite inbred lines was estimated using the ad hoc statistic delta K (ΔK) based on the rate of change in the log probability of the model between successive K values.

**Figure 5:**
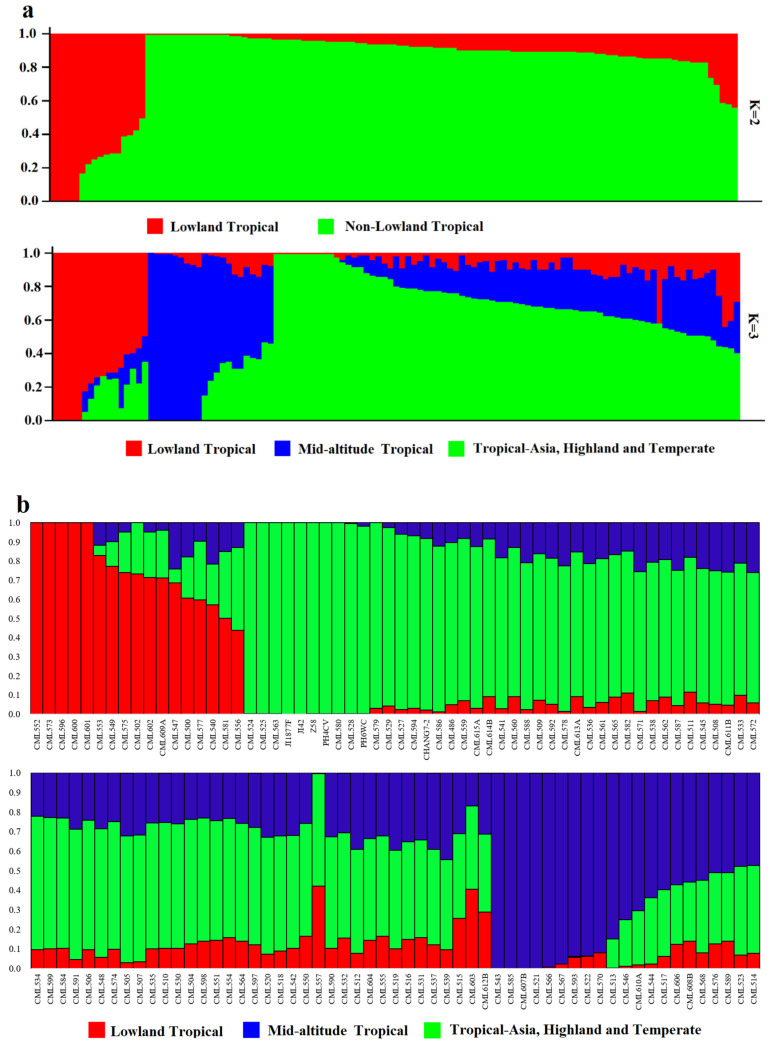
Population structure of 109 CMLs and 6 elite inbred lines. (**a**) Population structure of 109 CMLs and 6 elite inbred lines when K = 2 and K = 3. Each of the 538 individuals is represented by a thin vertical bar. (**b**) Bar plot of inbred admixture values, Q, which estimates the proportion of each individual’s genome that originated from each of the three inferred populations.

The number of best populations (K) inferred from a subset of 109 tropical germplasm and 6 elite inbred lines was estimated using a specific statistic, ΔK (Figure 4). The plot shows two peaks: one at K = 2 and the other at K = 3 (Figure 5). When K = 2, all inbred lines can be divided into two major categories: Latin American tropical lowland inbred lines and non-Latin American tropical lowland inbred lines. When K = 3, tropical germplasms from Africa form a separate group, while the remaining inbred lines constitute a third group, which includes key inbred lines from Asian tropical lowlands, high-altitude regions, and temperate zones. In the K = 2 classification, temperate germplasms were assigned to the non-Latin American tropical lowland cluster, whereas, at K = 3, they belonged to the third group. Dividing the population into three clusters allows for a more detailed examination of the genetic relationships between CML germplasm and temperate backbone inbred lines, providing additional insights into their genetic background and population structure.

### 2.5. Cluster Analysis

**Figure 6:**
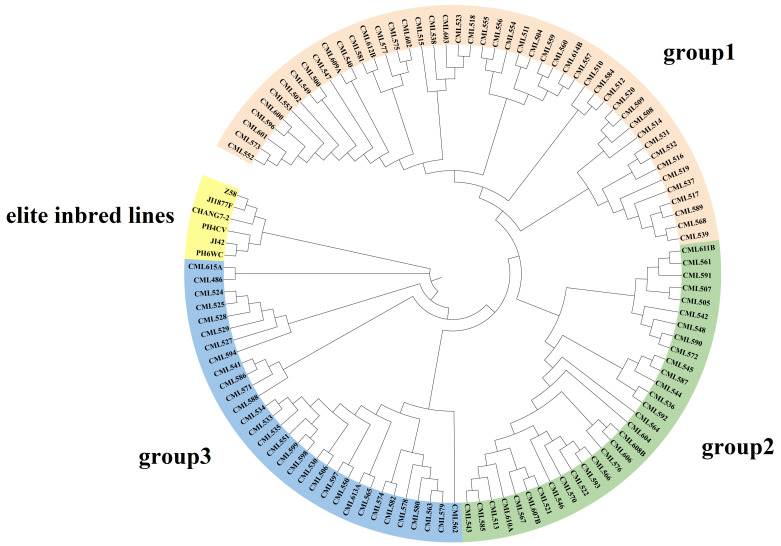
Neighbor-joining tree of 109 CMLs and 6 elite inbred lines based on the Rodgers modified genetic distance (MRD).

The results of cluster analysis indicated that 109 CML germplasms could be classified into three groups, which was similar to the results of structure analysis (Figure 6). Group 1 (No. = 41) consisted mainly of Middle Altitude/Sub-Tropical Africa germplasms (e.g., CML504, CML538, CML523, etc.) and Lowland Latin American germplasms (e.g., CML552-CML557). The clustering pattern of this group suggests that although these materials are from different continents, they show convergence in genetic structure, probably due to similar adaptive selection pressures (e.g., mild temperature, photoperiodic response). In addition, the dominance of African material in Group 1 (17/41) may reflect the long-term utilization of African adaptive germplasm by the CIMMYT breeding program. Group 2 (No. = 24) was centered on Middle Altitude/Sub-Tropical Africa (e.g., CML542 and CML590) and a small amount of Eastern Africa Highlands (EAH) germplasms (e.g., CML611B). Notably, although CML611B originated from highland environments, it clustered with mid-elevation material, possibly suggesting the presence of gene flow or the sharing of alleles for resilience (e.g., cold tolerance). Group 3 (No. = 32) had the most complex genetic composition and was dominated by Lowland tropical Asia material (e.g., CML562-CML582) and Lowland tropical Latin American lines (e.g., CML530-CML535). Temperate germplasm formed a distinct branch, indicating significant genetic differentiation between it and tropical germplasm.

Compared with the results of the structure analysis, the CMLs in group 1 are mainly distributed in the lowland-tropical and mid-altitude subgroups. The inbred lines included in the CML552-CML540 branch within group 1 originate from tropical lowlands and have the highest proportion of the genome originating from tropical lines; they are distributed in the lowland-tropical subgroup. The other inbred lines in group 1, although containing a relatively high proportion of tropical inbred components, have a higher proportion of genetic genome from temperate and subtropical sources, such as CML557, CML603, and CML612B. The materials in group 2 are primarily distributed in the mid-altitude, Tropical-Asia, highland, and temperate subgroups. Among them, the CML543-CML608B branch is located in the mid-altitude subgroup, while the others are distributed in the Tropical-Asia and highland and temperate subgroups. The remaining CMLs in group 2 also contain a certain proportion of subtropical genetic origins and a higher proportion of temperate origins.

### 2.6. Principal Component Analysis (PCA)

**Figure 7:**
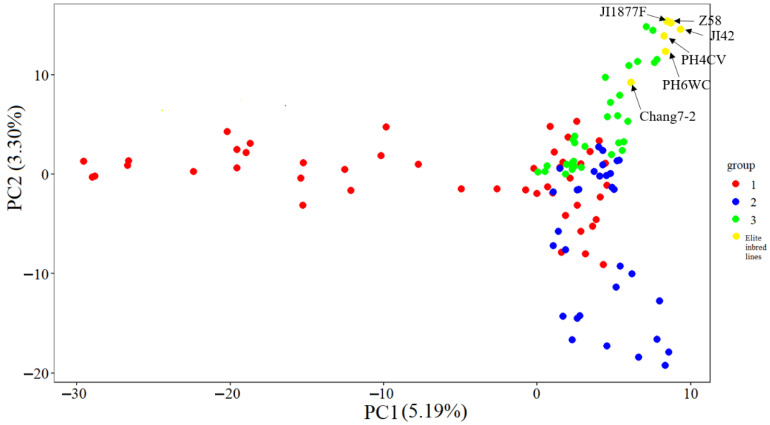
Scatter plot of the first two principal components (PC1 and PC2) for the 109 CMLs and 6 elite temperate inbred lines.

The results of the PCA clearly revealed the genetic structure characteristics of the CML germplasms (Figure 7). A two-dimensional scatter plot based on the first two principal components showed that the 115 materials (including 109 tropical germplasm and six temperate elite inbred lines) could be clearly classified into three genetic clusters labeled as red (cluster 1), blue (cluster 2), and green (cluster 3). The three genetic clusters showed significant segregation patterns in their spatial distribution: cluster 1 (red) was mainly distributed in the PC1 positive semi-axis region, cluster 2 (blue) was concentrated in the PC1 negative semi-axis and PC2 positive semi-axis quadrants, while cluster 3 (green) was significantly clustered in the PC1 negative semi-axis and PC2 negative semi-axis quadrants (*p* < 0.001). The compact distribution of individuals within each cluster and the obvious genetic gaps between clusters indicate significant population stratification and high intra-group homogeneity. This distribution pattern was highly consistent with the structure analysis (ΔK = 3) and the phylogenetic tree topology, which together confirmed that the test material had a clear genetic structure.

### 2.7. Analysis of Population Genetic Differentiation

The results of population genetic differentiation analysis (Table 2) showed that the genetic differentiation among the three subgroups within tropical germplasm (groups 1–3) was relatively low (F_ST_ = 0.043–0.052), indicating gene flow or shared ancestral polymorphism among them. Among them, the Fst values of group 2 and group 3 are relatively small, indicating that the genetic relationship between these two groups is relatively close. In contrast, significant differentiation was observed between tropical and temperate germplasms (F_ST_ = 0.117–0.167). Among these, group 1 exhibited the highest level of differentiation from temperate germplasms (F_ST_ = 0.167), while group 3 showed relatively lower differentiation from temperate germplasms (F_ST_ = 0.117).

Further analysis revealed that the F_ST_ value between the tropical hybrid group and the temperate germplasm was 0.131, the level of differentiation within the CML germplasms was weak, whereas the level of differentiation between the CML germplasms and the temperate germplasms reached a moderate to high level. The relatively low Fst between group 3 and temperate germplasms predicts greater genetic similarity.

**Table 2:** Pairwise Wright’s fixation index (F_ST_) values for the five germplasm groups.

	Group 1	Group 2	Group 3	Temperate Germplasm	Tropical Germplasm
Group 1	-				
Group 2	0.052	-			
Group 3	0.048	0.043	-		
Temperate germplasms ^a^	0.167	0.148	0.117	-	
Tropical germplasms ^b^	-	-	-	0.131	-

a. Six elite temperate lines. b. 109 CML germplasms.

## 3. Discussion

The Huanghuaihai region is one of China’s vital grain-producing areas. In recent years, the increasing frequency of biotic and abiotic stresses due to climate change has posed a threat to maize production in China [8,9]. Tropical germplasms represent an untapped reservoir of beneficial alleles for temperate maize enhancement, which must be characterized and utilized to enable future genetic gains, for example, *lycopene epsilon cyclase* for the provitamin A content in the maize kernel [19], and ZmRtn16 for drought resistance [6]. Introducing tropical germplasms to broaden the genetic diversity of elite inbred lines and enhance their adaptability serves as a crucial strategy to address this challenge. CIMMYT plays a significant role in tropical corn breeding. In recent years, an increasing number of CIMMYT germplasms have been introduced into China, especially in southwest China, as its climate is tropical or subtropical [16,20]. We have imported the newly released CML germplasms and attempted to utilize them for improving elite inbred lines in our region. The classification of their genetic diversity into heterotic groups facilitates the selection of suitable inbred lines for improvement.

### 3.1. Characterization of Genetic Diversity of Tropical Maize Germplasm and Its Breeding Value

Tropical germplasms are generally considered to possess greater genetic diversity than temperate germplasm [13,21]. Our results are consistent with those of previous studies. In this study, we found that tropical maize germplasms exhibited rich genetic diversity, and their average LD decay distance (97.16 Kb at r^2^ = 0.1) was significantly shorter than that of temperate materials [16,20], a feature closely related to the complex and diverse ecological environment and long-term natural selection in the tropics. The significant difference (*p* < 0.01) in LD decay distance among different chromosomes reflected the uneven distribution of genomic recombination rates, with chromosome 5 decaying the slowest (144.3 Kb) and chromosome 2 decaying the fastest (18.9 Kb). From a population genetics perspective, shorter LD decay distances significantly improve the resolution of genome-wide association analysis (GWAS), allowing trait-marker associations to be localized to more precise genomic regions. This property is particularly beneficial for fine localization of quantitative trait loci (QTL), as weaker LDs mean that associations between markers and causal variants are more specific [22].

### 3.2. Genetic Structure of CIMMYT Lines

CIMMYT’s heterotic group classification remains ambiguous [5,10]. Previous studies have indicated that the unclear delineation of its heterotic groups adversely affects tropical maize breeding and the utilization of CIMMYT germplasm for improving temperate inbred lines. Predominant racial origin of the source population and combining ability with established heterotic testers as either Tuxpeño (group A; e.g., population 21) or non-Tuxpeño (group B; e.g., population 32). However, studies indicate that the genetic divergence between these two populations is not significant [5,10,15]. DNA markers can be used not just to aid selection but also to help assign lines to heterotic groups and avoid making breeding crosses between lines that are supposed to belong in different heterotic groups [7,8,9,20]. The CIMMYT germplasm is usually classified based on adaptability into tropical, tropical lowland, and tropical highland [5,10]. There is germplasm exchange among different regions, which also occurs in CIMMYT in Latin America, Asia, and Africa. In this study, 109 CIMMYT germplasms were clustered according to ecological zones and origin. The tropical lowland and tropical mid-altitude germplasms basically clustered together. From the perspective of origin, inbred lines from Latin America, Africa, and Asia also basically clustered together. This is similar to previous research results. However, some inbred lines are from the same adaptability region but different areas. This exchange promotes the breeding of tropical germplasm and expands the genetic diversity of maize in this region.

### 3.3. Genetic Characterization of Temperate Elite Inbred Lines and Breeding Utilization of Tropical Germplasm

Tropical germplasms possess high genetic diversity and harbor numerous superior genes [6,13,19,21]. Tropical and subtropical lines were found to contain many rare alleles in past studies [23] and are an important resource to find new functional alleles of desired traits and can be used to broaden the genetic base of maize breeding populations or to find sequence variation for targeted introgression into temperate breeding lines [5,6,16]. These rare genes represent an untapped treasure trove. The Huanghuaihai region of China is one of the country’s primary maize-growing areas, where both biotic and abiotic stresses are prevalent. Ear rot, stalk rot, and leaf spot are the major diseases affecting maize in this region [8,9]. Due to the simultaneous occurrence of rainfall and heat, drought in the early season and flooding in the late season are common occurrences in this region. Simultaneously, the distribution of southern rust has been steadily shifting northward, transforming regions previously unaffected into primary infection zones. Concurrently, the implementation of winter maize wheat rotation systems has imposed stringent requirements on corn maturity dates within this area [8,9]. Increasing the genetic diversity of maize in this region and continuously introducing genes resistant to biotic and abiotic stresses into the elite inbred lines is the solution to the above problems.

Evidence from tests of genetic divergence using F_ST_ between heterotic groups, temperate germplasm, and population 3 shows the closest genetic distance, followed by subpopulation 2. Six elite inbred lines can be divided into two groups. These two groups play an important role in corn breeding in the Huanghuaihai region. To preserve the genetic diversity and heterosis potential of these two groups to the greatest extent, it is recommended to improve the corresponding local groups using groups 2 and 3, respectively. However, the classification of heterotic groups using molecular markers is the first step for improving temperate germplasm with tropical sources. Nevertheless, combining the ability test and phenotypic performance identification remains indispensable [20]. In the Huang-Huai-Hai region, inbred tropical maize lines typically exhibit photoperiod sensitivity, which in turn leads to delayed silking and pollen shedding, or even complete sterility. This phenomenon is more pronounced in lowland tropical inbred lines compared to those from subtropical and highland tropical. Additionally, some tropical germplasms exhibit significant phenotypic differences between tropical and temperate regions. Thus, evaluating their phenotype and combining ability in advance is essential for their utilization in temperate germplasm improvement.

In this study, significant genetic differentiation between temperate elite inbred lines and tropical germplasms was revealed by population genetic analysis (F_ST_ = 0.117–0.167), confirming the genetic characterization of the two as independent gene pools. This differentiation stems mainly from long-term artificial selection, different domestication histories, and differences in ecological adaptations. Notably, the Asian tropical germplasm (group 3) showed relatively low genetic distance (F_ST_ = 0.117) from temperate inbred lines, suggesting that it may be used as a bridge germplasm for genetic improvement of temperate maize. Based on the results of population structure (ΔK = 3) and F_ST_ analysis, the hybrid dominance group can be optimized.

## 4. Materials and Methods

### 4.1. Plant Material

A total of 109 representative tropical maize germplasm accessions from CIMMYT (Mexico) were collected to assess genetic diversity and population structure (Table 3). These 109 CMLs were categorized into 7 groups based on their geo-ecological adaptations, covering different ecological regions such as Africa, Latin America, and Asia. The most numerous subgroups were Middle Altitude/Sub-Tropical Africa (46), Lowland tropical-Latin America (20), and Lowland tropical-Asia (13). Among them, Middle Altitude/Sub-Tropical Africa contained the largest number of inbred lines (e.g., CML589, CML539, CML591, etc.). In addition, there are 8 varieties in the highlands (e.g., CML594 and CML527). Additionally, six temperate elite inbred lines (e.g., PH6WC, PH4CV), which are commonly used in China as indicator lines for the classification of hybrid heterotic groups, were included as an outgroup for comparative analysis. The six elite inbred lines adopted in this study can be classified into two main groups: One is the temperate inbred maize lines and their derivatives, originating from the United States, including PH6WC, PH4CV, and Ji42. The other is the inbred lines formed based on the continuous improvement of local farm varieties and other germplasm resources, including CHANG 7-2, Ji1877F, and Z58.

### 4.2. SNP Genotyping

One hundred and nine tropical germplasm and six temperate inbred maize lines were planted at Dishang Experimental Station, Shijiazhuang, Hebei Province, China. For all of the maize lines tested in this study, leaf samples bulked from 15 plants of each line were used for DNA extraction with a CTAB procedure [24].

The extracted DNA was sent to the MolBreeding company for 10 K BeadChip sequencing, which uses Genotyping By Target Sequencing (GBTS) [25]. This technology realizes in-depth resequencing through specific enrichment of the target loci. The specific process is as follows: Firstly, use restriction endonuclease to fragment the DNA, and take 500 ng of fragmented DNA to construct the sequencing library. Then, add specific probes and hybridization reagents to carry out the hybridization reaction. After the completion of the hybridization, remove the unbound fragments through the elution step. Finally, carry out the PCR amplification and complete the construction of the hybridization-captured library. Due to the fact that the chips are developed based on germplasms widely used in China and the proportion of temperate germplasms is relatively high, SNP calling was performed with B73 RefGen_v5 as the reference genome to generate a comprehensive genotype collection.

### 4.3. SNP Characteristics

Marker summary statistics were calculated for 10,005 markers using R version 4.3.0, including heterozygosity [26], percentage of missing data, and Shannon–Wiener index for each locus. The Shannon–Wiener index (H) for each marker was calculated as follows:

H = −∑[(p*_i_*) × ln(p*_i_*)],

where p*_i_* is the allele frequency of the i th allele.

### 4.4. Linkage Disequilibrium (LD)

Using TASSEL 5.2 software [27], chromosomes were segmented in a 50 kb window, and the LD values of pairs of SNPs were calculated by Pearson correlation coefficient (r^2^), and the average LD of each segment was assessed [28]. In genome-wide and subgroup analyses, the LD attenuation level was calculated based on the r^2^ values of the 10,005 SNPs on each chromosome, and the average LD attenuation distance of ten chromosomes was determined using an r^2^ = 0.1 threshold. LD attenuation distances and compare the differences between groups [18]. Window analysis was performed using a 50 kb sliding window (step size 50 kb) to calculate the mean LD values between loci.

### 4.5. Population Structure Analysis

Population structure was analyzed using the Bayesian Markov Chain Monte Carlo (MCMC) program STRUCTURE 2.3.4 [29,30], using 10,005 SNPs to assign lines to subpopulations. Both the warm-up period and the MCMC replicate length were set to 10,000 iterations. A mixed model was used, assuming allele frequency correlation between groups. For each predefined number of gene clusters (k, ranging from 1 to 10), five independent runs were performed.

The ΔK method is a widely used statistical criterion for determining the optimal number of genetic subgroups (K) in population structure analysis [8]. Its core principle is to identify inflection points in the growth rate of the likelihood function by calculating the second-order rate of change in the log-likelihood values (ΔK) between neighboring values of K, thus avoiding subjective selection of K [17]. The formula is as follows:

$$\Delta K=\frac{\left|L^{\prime }\left(K+1\right)-2L^{\prime }\left(K\right)+L^{\prime }\left(K-1\right)\right|}{SD(L(K))}$$

included among these, L′(K) is the standardized log-likelihood value corresponding to K, and SD is the standard deviation. The peak of ΔK corresponds to the most significant population stratification signal, reflecting the true number of genetic clusters in the data.

### 4.6. PCA

Principal component analysis (PCA) was performed using genome-wide SNP data using TASSEL 5.2.80 software. The first 2 PCs with the highest contribution rate were selected for subsequent analysis, where the variance contribution rate of each PC was calculated as follows:

$$Variance\;explained\;by\;{PC}_{i}=\frac{\lambda_{i}}{\sum_{j=1}^{m}\lambda_{j}}$$

where λ_i_ is the eigenvalue of the ith PC and m is the total number of SNPs [31]. Subsequently, the first two principal components (PC1 and PC2) were visualized using the ggplot2 package in R (version 4.3.3) to show the sample distributions as scatter plots, colored by clustering results.

### 4.7. Cluster Analysis

The genetic distance between each inbred pair was estimated on 10,005 markers using Rodger’s Modified Genetic Distance (MRD). The MRD was calculated as follows:

$$MRD=\sqrt{\frac{1}{2m}\sum_{i=1}^{m}\sum_{j=1}^{a_{i}}{\left(p_{ij}-q_{ij}\right)}^{2}}$$

where p_ij_ and q_ij_ are the allele frequencies of the jth allele at ith locus in each pair of inbred lines, a_i_ is the number of alleles at the ith locus, and m is the number of loci [10]. A total of 109 tropical maize germplasms and 6 temperate elite inbred lines were sequenced using 10 K SNP chips. Cluster analysis of MRD matrices was performed using the neighbor-joining (NJ) method of MEGA 11 [32]. Dendrograms were generated and visualized using the R package ggtree 3.8.2 [33].

### 4.8. Population Distance

Pairwise Wright fixation indices (F_ST_) were calculated for each group using the VCF tools program 4.2 using 10,005 SNPs [34,35].

## Figures and Tables

**Table 1:** Average values of MAF, deletion rate, heterozygosity rate, and Shannon index of SNP sites in each chromosome.

	Minor Allele Frequency	Proportion Missing	Proportion Heterozygous	Shannon Index
Chr1	0.253	0.031	0.027	0.516
Chr2	0.258	0.033	0.027	0.523
Chr3	0.255	0.027	0.021	0.518
Chr4	0.262	0.031	0.03	0.531
Chr5	0.25	0.029	0.03	0.515
Chr6	0.257	0.034	0.029	0.525
Chr7	0.26	0.032	0.027	0.525
Chr8	0.267	0.032	0.03	0.532
Chr9	0.255	0.03	0.028	0.516
Chr10	0.251	0.035	0.034	0.51
Average	0.257	0.027	0.025	0.521

**Table 3:** Population structure and geographic origins of the tropical maize panel.

Geographic-Ecological Type	Representative Varieties (Count)
Middle Altitude/Sub-Tropical Africa (51)	CML504, CML505, CML506, CML507, CML508, CML509, CML510, CML511, CML513, CML514, CML520, CML521, CML522, CML523, CML536, CML537, CML538, CML539, CML540, CML541, CML542, CML543, CML544, CML545, CML546, CML547, CML548, CML559, CML560, CML568, CML566, CML567, CML570, CML571, CML572, CML584, CML585, CML586, CML587, CML588, CML589, CML590, CML591, CML592, CML593, CML609A, CML604A, CML608B, CML606, CML607B, CML610A
Lowland tropical-Latin America (20)	CML530, CML531, CML532, CML533, CML534, CML535, CML549, CML550, CML551, CML552, CML553, CML554, CML555, CML556, CML557, CML573, CML574, CML575, CML576, CML577,
Lowland tropical (11)	CML500, CML502, CML515, CML516, CML596, CML597, CML598, CML599, CML600, CML601, CML602
Subtropical tropical (6)	CML486, CML512, CML517, CML518, CML519, CML603
Highland tropical (6)	CML524, CML525, CML527, CML529, CML528, CML594,
Lowland tropical-Asia (13)	CML562, CML563, CML564, CML565, CML578, CML579, CML580, CML581, CML582, CML612B, CML613A, CML614B, CML615A
Highland tropical Eastern Africa (2)	CML561, CML611B,
Temperate Germplasm (6)	PH6WC, PH4CV, Ji42, CHANG 7-2, Ji1877F, Z58

## Data Availability

The datasets generated during and/or analyzed during the current study are available from the corresponding author upon reasonable request.
